# Lectin-like transcript 1 as a natural killer cell-mediated immunotherapeutic target for triple negative breast cancer and prostate cancer

**DOI:** 10.20517/2394-4722.2019.29

**Published:** 2019-12-17

**Authors:** Yuanhong Sun, Joseph D. Malaer, Porunelloor A. Mathew

**Affiliations:** Department of Microbiology, Immunology and Genetics, University of North Texas Health Science Center, Fort Worth, Texas 76107, USA.

**Keywords:** Natural killer cell, lectin-like transcript 1, CLEC2D, CD161, breast cancer, prostate cancer, immunotherapy

## Abstract

Breast and prostate cancer are the leading causes of death in females and males, respectively. Triple negative breast cancer (TNBC) does not express the estrogen receptor, progesterone receptor, or human epidermal growth factor receptor 2, resulting in limited treatment options. Androgen deprivation therapy is the standard care for prostate cancer patients; however, metastasis and recurrence are seen in androgen-independent prostate cancer. Both prostate and breast cancer show higher resistance after recurrence and metastasis, which increases the difficulty of treatment. Natural killer (NK) cells play a critical role during innate immunity and tumor recognition and elimination. NK cell function is determined by a delicate balance of inhibitory signals and activation signals received through cell surface receptors. Lectin-like transcript 1 (LLT1, CLEC2D, OCIL) is a ligand of NK cell inhibitory receptor NKRP1A (CD161). Several studies have that reported higher expression of LLT1 is associated with the development of various tumors. Our studies revealed that TNBC and prostate cancer cells express higher levels of LLT1. In the presence of a monoclonal antibody against LLT1, NK cell-mediated killing of TNBC and prostate cancer cells were greatly enhanced. This review highlights the potential that using monoclonal antibodies to block LLT1 - NKRP1A interactions could be an effective immunotherapeutic approach to treat triple negative breast cancer and prostate cancer.

## INTRODUCTION

Breast cancer is one of the most serious diseases influencing the health of women, whereas prostate cancer threatens the health of men^[1]^. In the 2019 Cancer Statistics report by Siegel *et al.*^[2]^, it was estimated that more than 170,000 men will be diagnosed with prostate cancer in the United States in 2019. Triple negative breast cancer (TNBC), a subtype of breast cancer, has the poorest prognosis and highest rate of recurrence and metastasis^[3]^. Cancer recurrence and metastasis is the primary cause of cancer-related deaths and resistance to previous treatments is commonly seen. The critical functions of the immune system in cancer have been known for decades, but the transition to immunotherapy as a major method of cancer treatment has only occurred within the past few years. After the immunoediting hypothesis was suggested, the role of immune cells in cancer cell elimination, tolerance, and escape has been recognized^[4]^. Although our immune system utilizes both innate and adaptive immune cells to recognize and eliminate abnormal cells, in many situations, cancer cells are still able to avoid immune surveillance. Cancer cells can adopt several mechanisms to evade immune surveillance; one mechanism is the upregulation of inhibitory signal pathways of immune cells to inhibit the immune response, such as upregulation of PD-L1 in TNBC^[5]^. Lectin-like transcript 1 (LLT1) is a natural killer (NK) cell inhibitory ligand that has been described to contribute to the immunosuppressive properties of glioblastoma, prostate cancer, and triple negative breast cancer^[6–9]^. This review highlights breast and prostate cancer, the function of NK cells, and NK cell-based immunotherapy. Additionally, this review explores LLT1 as a potential immunotherapeutic target for breast and prostate cancer elimination by NK cells.

## BREAST CANCER AND PROSTATE CANCER

In 2019, there will be approximately 270,000 women diagnosed with breast cancer and 170,000 men diagnosed with prostate cancer in the United States^[2]^. Unlike other cancers, breast cancer and prostate cancer are primarily gender specific in females and males, respectively. According to the expression level of estrogen receptor, progesterone receptor, and human epidermal growth factor receptor 2 (HER2), breast cancer is classified into the subtypes luminal A, luminal B, HER2 positive, and TNBC^[10,11]^. Compared with the other subtypes of breast cancer, TNBC has a relatively worse prognosis owning to lack of recognizable molecular targets and high level of heterogeneity^[10,12–14]^. Conventional treatments, including chemotherapy and radiotherapy, are still the primary way to treat TNBC patients. Beneficial results are observed when patients first begin chemotherapy; however, resistance to the chemotherapy and recurrence have been shown after later treatments^[15]^. *BRCA1*, *BRCA2*, *ATM*, and *TP53* are essential genes in the DNA damage response signal pathways^[16]^. Studies suggest mutations in these genes are found in TNBC^[16,17]^. In TNBC, critical DNA damage response gene mutations lead to genomic instability, and a higher probability to produce neoantigens, which are termed “non-self” to differentiate from “self”^[18]^. These neoantigens offer promising targets for immunotherapy.

In the United States, prostate cancer is the second leading cause of cancer-related death in males. Due to the vital role of the androgen receptor in the development of prostate cancer, androgen deprivation therapy has become the standard treatment for prostate cancer^[19–22]^. Prostate cancer recurrence is usually androgen independent, which is termed castration-resistant prostate cancer^[23,24]^. Therefore, new treatments are required for castration-resistant prostate cancer.

## NK CELL FUNCTION

NK cells are an indispensable component of immune cells, but their role in immunotherapy has only been considered in recent years. NK cells were first suggested in tumor immunosurveillance due to studies that showed people with higher incidence of cancers have defective NK cell functions caused by gene deficiency^[25,26]^. Additionally, tumor growth and metastasis were also observed in NK mutant mice or after blocking NK cell activity by antibodies^[27,28]^. Two types of receptors are expressed on NK cells: inhibitory receptors and activation receptors. Natural cytotoxicity receptor family, killer cell lectin-like receptor, and CD16 comprise the majority of activation receptors expressed on NK cells^[29,30]^. The common characteristic of these NK cell activation receptors is having a cytoplasmic immunoreceptor tyrosine-based activation motif to mediate the activation signals. Unlike NK cell activation receptors, inhibitory receptors such as killer cell immunoglobulin-like receptors and the heterodimer CD94-NK group 2A recognize and bind to self MHC class I molecules and the inhibitory signals are mediated via cytoplasmic immunoreceptor tyrosine-based inhibition motifs^[31]^. In contrast with CD8^+^ T cells, activation of NK cells does not require antigen presenting cell priming or MHC restriction^[32]^. NK cell activation is regulated by the balance from activation and inhibitory receptor mediated signaling^[33]^. Therefore, through multiple, simultaneous complex signaling pathways, NK cells recognize and kill a broad range of tumor cells. For example, tumor specific antigen interaction with activation receptors on NK cells, accompanied with lack of co-engagement of inhibitory receptors, will lead to secretion of perforin and granzyme from NK cells to target tumor cells^[34]^. Additionally, it has been reported that some tumor cells have spontaneous loss of MHC class I expression as a mechanism for CD8^+^ T cell escape. NK cells, which do not require antigen presentation, are able to recognize and kill MHC class I low tumor cells^[35]^.

## NK CELLS AND IMMUNOTHERAPY

Antibody dependent cell-mediated cytotoxicity (ADCC) is another primary function of NK cells and is currently being investigated to be used in NK cell-mediated immunotherapy. NK cells use the CD16 (Fc_γ_RIII) receptor to bind with the Fc portion of antibodies bound to specific antigens on target cells and induce NK cell cytotoxicity^[36]^. Monoclonal antibodies (mAb) can also be used to block the interaction between ligands and receptors on NK cells. We have shown that activating NK cells via surface receptor CS1 (CD319, SLAMF7) enhanced the ability of NK cells to kill various tumor cells^[37,38]^. Several other studies showed that CS1 is overexpressed on multiple myeloma (MM) and a humanized anti-CS1 mAb (Elotuzumab/Empliciti) was approved as a break through drug for treatment for MM patients^[39,40]^. Our lab has found overexpression of LLT1 ligand on TNBC and prostate cancer, which interacts with the NK receptor NKRP1A (CD161) and inhibits NK cell cytotoxicity^[9,41]^. Thus, blocking inhibitory signals to NK cells using monoclonal antibodies to LLT1 could enhance the lysis of prostate cancer and TNBC cells by NK cells^[9,41]^.

Checkpoint inhibitors, which are traditionally used to promote CD8^+^ T cell function, have also been demonstrated to effect NK cell function. More specifically, NK cells express PD-1 and binding to PDL-1 on target cells results in inhibition of NK cell function and more aggressive tumors. Blockade of PD-1 and PDL-1 with mAb results in the strong NK cell responses required for full immunotherapeutic effect^[42]^.

In addition to checkpoint inhibitors, CARs (chimeric antigen receptor) can be engineered in NK cells or NK cell lines. Binding of a CAR to a tumor associated antigen leads to a strong activating signal, which triggers NK cell cytotoxicity^[43]^. Currently, there are many clinical trials taking advantage of CAR NK cells. One such trial (NCT03056339) utilizes a CAR specific for the tumor associated antigen CD19 coupled to the costimulatory domains of CD28 and the zeta chain of the TCR/CD3 complex. Additionally, the retroviral vector expresses IL-15 for optimal NK cell activation and the inducible caspase 9 (iC9; iCasp9) suicide gene.

## LLT1 AND NKRP1A

LLT1, also known as CLEC2D (C-type lectin domain family 2 member D), OCIL (Osteoclast inhibitory lectin), and CLAX (Lectin-like NK cell receptor), belongs to the C-type lectin-like receptor superfamily and was first described and cloned by our lab in human NK cells^[44]^. Cross-linking of the LLT1 receptor on NK cells induces the production of IFN-_γ_ without activation of the cytolytic pathway^[45]^. Our lab, confirmed by others, also identified the NK cell inhibitory receptor NKRP1A (CD161) as the ligand for LLT1 and implicated that LLT1 expressed on other cells could inhibit NK cell function^[46,47]^. LLT1 is encoded by the *CLEC2D* gene, which produces five different isoforms through alternative splicing. Isoform 1 of LLT1 interacts with the NKRP1A (CD161) receptor. Isoforms 2 and 4 stay in the endoplasmic reticulum and associate with LLT1 to form homodimers or heterodimers. Unlike LLT1 expressed on the cell surface, Isoforms 5 and 6 are soluble forms of LLT1^[48,49]^. As a C-type lectin-like receptor, LLT1 is composed of three domains: a transmembrane domain, a stalk region, and the extracellular carbohydrate recognition domain, which is responsible for recognition^[7]^. B cells, NK cells, T cells, and activated dendritic cells also express LLT1 on the cell surface^[48,49]^. The crystal structure of LLT1 demonstrated it is highly glycosylated and when it forms a homodimer at the cell surface it serves as a ligand for the NKRP1A receptor on NK cells^[46,47,50,51]^. NKRP1A is encoded by *KLRB1* and CD4^+^ T cells, invariant NKT cells, and _γδ_-T cells have also been shown to express NKRP1A^[49]^. In mice, *CLEC2D* encodes the protein Ocil/Clr-b, which interacts with NKR-P1B/D^[52–54]^.

## LLT1 AS AN IMMUNOTHERAPEUTIC TARGET FOR BREAST CANCER AND PROSTATE CANCER

**Figure 1.**
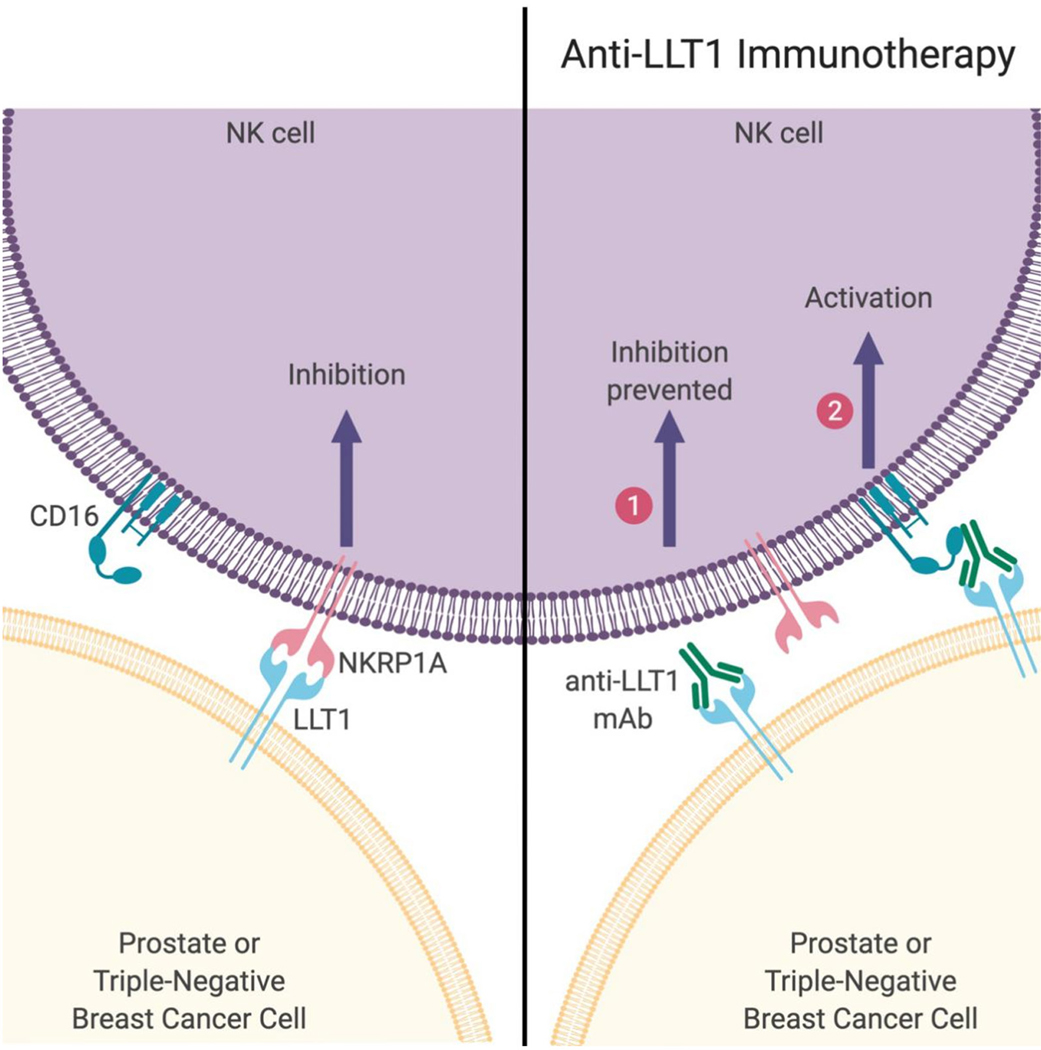
Activation of NK cell using anti-LLT1 monoclonal antibody. (Left) Prostate and triple-negative breast cancer cells express LLT1 which can bind to NKRP1A on NK cells leading to inhibition of NK cell function. (Right) However, in the presence of anti-LLT1 antibodies, NK cells can be activated via two mechanisms: (1) blocking of LLT1 prevents inhibitory signal transmission; and (2) activation when NK cell receptor CD16 binds to anti-LLT1 antibody bound to LLT1 on cancer cells. Image created using BioRender (www.BioRender.com). NK: natural killer; LLT1: lectin-like transcript 1; mAb: monoclonal antibody

NK cells, B cells, T cells, and activated dendritic cells express LLT1^[46,48]^. Monoclonal antibody blocking of LLT1 could enhance the IFN-_γ_ production of NK cells, but no differences in cytotoxicity have been seen^[45]^. Increased expression of LLT1 was observed after Toll-like receptor induced activation of B cells and dendritic cells, which led to NK cell inhibition via LLT1 - NKRP1A interaction^[49]^. Inhibition of NK cell function through LLT1 has been observed on human malignant glioma cells, and immune crosstalk with B cells and monocyte-derived dendritic cells^[8,49,55]^. Identification of a specific marker to enhance the ability of NK cells to recognize TNBC cells and prostate cancer cells provides a promising way for clearance of cancer cells. Previously, our lab studied the expression of LLT1 on TNBC and prostate cancer, and the results showed higher expression of LLT1 on TNBC cells compared to normal cells. Monoclonal antibody blocking of LLT1 enhanced NK cell cytotoxicity and TNBC cells lysis^[9]^. In addition to higher surface expression of LLT1 on TNBC cells, our studies also demonstrated higher intracellular and surface expression of LLT1 in the prostate cancer cells compared with normal prostate cells. Blocking the interaction between LLT1 and NKRP1A increased NK cell cytotoxicity against prostate cancer cells^[41]^. In addition to blocking the inhibitory signal, anti-LLT1 mAb may activate ADCC (antibody dependent cell mediated cytotoxicity) function of NK cell against breast cancer and prostate cancer [Figure 1]. Pasero *et al.*^[56]^ showed that highly effective NK cells are associated with better prognosis in metastatic prostate cancer patients. Collectively, we demonstrate that monoclonal antibodies to enhance NK cell activation may provide a promising treatment to target and potentially prevent breast and prostate cancer metastasis. Santos-Juanes’ newest study showed higher LLT1 expression was contributed to the risk of neck cutaneous squamous cell carcinoma (cSCC) nodal metastasis, which implicates LLT1 may also be a potential target to block cSCC nodal metastasis^[57]^. Similar results have also been seen in mice; inhibition of NKRP1B: Clr-b axis could enhance the NK cell-mediated immune surveillance to oncogenic transformation^[52]^. Due to the important role of LLT1 in tumor progression and metastasis, monoclonal antibody blocking of LLT1 to enhance NK cell cytotoxicity may provide a novel treatment to prevent tumor metastasis.

## CONCLUSION

Tumor recurrence and metastasis is associated with chemotherapy resistance, which greatly enhances the mortality in breast and prostate cancer^[3,58]^. NK cell-mediated immunotherapy could provide specific recognition of tumor cells and overcome the resistance from chemotherapeutic drugs. LLT1, an inhibitory ligand, is widely expressed on several cancer cell lines. Our lab has demonstrated higher expression of LLT1 on TNBC and prostate cancer cells and increased lysis of cancer cells after blocking LLT1 with monoclonal antibodies. These results suggest LLT1 may offer a potential target for breast and prostate cancer treatment.
